# The Impact of Time Spent in Natural Outdoor Spaces on Children’s Language, Communication and Social Skills: A Systematic Review Protocol

**DOI:** 10.3390/ijerph191912038

**Published:** 2022-09-23

**Authors:** Steph Scott, Tonia Gray, Jenna Charlton, Sharon Millard

**Affiliations:** 1 Shropshire Community Health NHS Trust, Coral House, Longbow Close, Shrewsbury SY1 3GZ, UK; 2 School of Nursing and Midwifery, The University of Birmingham, Edgbaston, Birmingham B15 2TT, UK; 3 Centre for Educational Research, Western Sydney University, Sydney, NSW 2751, Australia; 4 School of Education, Newcastle University, Newcastle upon Tyne NE1 7RU, UK; 5 Michael Palin Centre for Stammering, 13-15 Pine Street, London EC1R 0JG, UK

**Keywords:** children, communication, language development, social skills, nature, outdoor play

## Abstract

There has been increasing interest over the past decade with regard to the health and wellbeing implications of time spent outdoors in nature for children. Universal systematic reviews of evidence report benefits to physical health, social-emotional mental health and wellbeing, cognition and academic learning. Internationally, there is indicative evidence to suggest outdoor engagement with nature may also impact children’s language and communication skills, skills that are critical to development, education, social relationships and life opportunities. Yet, at present such evidence has not been synthesised. Despite evidence for the benefits of the outdoors, the amount of time children are spending outdoors is in rapid decline, and has been further exacerbated by the COVID-19 pandemic. Alongside this are increasing numbers of children starting primary education with significant speech, language and communication needs (SLCN) which remain persistent over time. With established wide-reaching benefits of nature to children’s physical and mental health and psychological development, there is a need to further explore the more specific impacts of the natural environment on children’s language, communication and social skills, which could provide a unique opportunity to consider nature as a universal public health intervention for SLCN. The current review will aim to synthesise existing qualitative and quantitative evidence of the impact of time spent in natural outdoor spaces on the language, communication and social skills of 2–11-year-old children. Literature will be searched across seven databases and considered for inclusion against inclusion and exclusion criteria. Potential implications of the review include informing public health practice and policy for child development and education, informing priorities for speech, language, and communication interventions, and providing directions for future international research.

## 1. Introduction

The myriad benefits that humans accrue from time spent in natural outdoor spaces are well documented and include improved physical health, mental health and wellbeing, and creativity [1,2,3,4,5,6,7,8,9]. There is also growing evidence documenting the numerous benefits to child development from spending time outdoors [10,11,12,13] including physical and mental health impacts but in addition to these are indicative benefits to children’s cognitive, academic attainment, and physical activity levels [14,15,16,17], outcomes which are also documented across systematic reviews of the literature [11,18,19,20]. Less referenced in the literature are the impacts of nature on children’s language, communication and social development. Early language and communication skills in particular are pivotal to children’s non-physical development and life opportunities; they are essential to educational engagement and achievement, social relationships and future employment prospects [21,22]. However, more than 1.4 million children in England have Speech, language and communication needs (SLCN) [23] and it is estimated that between 5% and 8% of all children in the UK have early language difficulties, a figure that rises to over 20% for those growing up in low-income households [24,25]. SLCN are also identified as the most common type of Special Educational Need (SEN) in England, at 25.1% of pupils [26]. Currently there is no systematic review of the impacts of nature on children’s language development although there is some evidence to suggest the outdoor environment has positive impacts on children’s language and communication [27] lexical diversity, and fluency of language learning [28].

Yet, despite a growing evidence-base for the wide-reaching beneficial effects of nature on child development, the amount of time children are spending outdoors is in rapid decline; in the UK, today’s children are spending half the time playing outside than their parents did [29], a trend echoed internationally [30]. Barriers such as parental concerns around safety, access to natural green spaces (in particular inequality between urban and rural areas) and children’s tendency towards screen play indoors, are reducing children’s contact with the natural world around them [18,31,32]. Such barriers have been further exacerbated by the COVID-19 pandemic, and there is clear evidence indicating that during this time children around the world significantly reduced their outdoor engagement [33] and physical activity levels [34,35], and increased their screen time [36] and sedentary behaviour [37,38]. Furthermore, although the effects of COVID-19 on children have been multifaceted, it is less clear how the pandemic has impacted children’s speech and language development. These impacts may not be fully realised for some time, although early evidence from primary schools in England indicates children who started school in 2020 required additional support with language and communication compared to previous years [39,40]. 

Understanding the impacts of the natural environment on children’s language, communication and social skills could provide a unique opportunity to consider nature as universal public health intervention. Parents, educators, clinicians and policy makers require robust evidence to help shape their decisions around prioritising children’s time outdoors. Significantly, the National Institute for Health Research [41] has recently called out for research proposals exploring which interventions lead children and young people to spend more time outdoors in ways that improve their health and reduce health inequalities. Quite clearly the pressing demand for research in this area is growing in significance and priority.

***The Current Study:*** The current study will address a gap in the evidence-base exploring the effects of time spent in natural outdoor spaces on children’s language, communication and social skills. While there are some individual studies exploring these impacts, there has not as yet been a systematic approach to reviewing the literature on this topic. If synthesized systematically, outcomes may have the potential to influence UK society’s approach to prioritizing children’s time and space outdoors for the purposes of speech and language interventions, outdoor education and public health.

**Background:**

***The impacts of the outdoors on children’s language, communication, and social development:*** There is increasing evidence that demonstrates the benefits of nature to a child’s language, communication and social development. For example, children use five times more words when playing outdoors compared to indoors [42]. They have a greater sense of wellbeing, higher self-esteem and confidence and they laugh more [43]. Their attainment in English SATs and language skills on school entry are significantly higher [44]. Research by O’Brien [27] demonstrates specific improvements in children’s language and communication skills, especially for children who do not have access to nature as part of their everyday lives and makes definitive links between outdoor nature play and children’s communication and social skills. Richardson & Murray [45] established correlations between outdoor nature play and an increase in children’s utterance quality. Research has also demonstrated that natural environments influence social interactions between parents and children by increasing connected, responsive communication [46].

***The importance of language for education:*** It is widely recognized that speech, language, and communication skills are central to children’s school readiness and ability to engage with the educational curriculum [22,47]. At every stage of education children with language difficulties fall behind their typically developing peers in academic achievement at attainment, from the Early Years, through the primary years and into secondary years [21]. The Communication Trust [48] has estimated that in some parts of the United Kingdom (UK), upwards of 50% of children are starting school with a SLCN. This prevalence has been reported as high as 84% in some areas of deprivation [49]. Almost all children with SLCN experience some difficulty with learning to read and write, placing them at increased risk of poor school readiness and inability to access the national curriculum [50]. According to Supporting Families in the Foundation [51], ‘children should start school healthy, happy, communicative, sociable, curious, active and ready equipped for the next phase of life and learning’. However, Ofsted [52] have reported that across the UK in 2014 only half of all children reached a good level of development by the age of five. For some children the picture was much worse, with over fifty local authorities reporting less than a third of children reached this level. The recent Bercow review found that in 2018 only 26% of young children with SLCN made expected academic progress in the Early Years Foundation Stage compared with 69% of all children, and only 15% of primary school pupils with SLCN achieved the expected levels in reading, writing and mathematics at the end of their primary school years compared with 61% of all children [23]. The impacts of poor language and communication on children’s education are therefore significant and long-term.

***Outdoor provision across the childhood years:*** In children’s early years the provision of the outdoor environment is seen as a space where developmental needs may be nurtured [53,54]. The Department for Education advocates for outdoor play as part of non-statutory curriculum in the early years [55] and exploring the natural world is a key early learning goal for children during these years [56]. It is recognised that throughout these preschool years, children learn very much through play, and therefore much of their outdoor engagements are play-based [56,57]. In contrast, we see less guidance across the curriculum about outdoor learning as children begin their primary school years, and instead greater emphasis is placed on the attainment of compulsory national curriculum subjects. Outdoor engagement across the primary years is often curriculum-based, including Physical Education but also core subject learning. Across the UK, the provision of outdoor -based curriculum delivery is increasing with promising evidence for beneficial effects on children’s learning and wellbeing [58]. It appears therefore that the nature of children’s engagement with the outdoors may change over time in-line with school stages and developmental competencies, with less structured, play-based and developmental provision in the early years, and more structured curriculum-based provision in the primary years. However, evidence for the comparative effects of different types of provision on language and social outcomes is scarce, and it is also unclear for which age range nature has the most beneficial effects.

***The impacts of limited outdoor engagement***: Contributing to poor developmental outcomes for children are factors such as the amount of indoor time children are increasingly experiencing [12], the amount of screen time now accessed by young children [59], and of primary importance to this study is research relating to a child’s contact with the natural world [60]. A government funded survey in England [61] suggests children spend less time outdoors than prison inmates. They also found that in the 12 months leading up to the study, more than one in nine children had not set foot in a forest, on a beach or any other natural environment. The survey showed that families in England with low socio-economic status (SES), and minority ethnic households were markedly less likely than white children and those from higher income households to visit urban or rural wild places. These sub-groups are among others referred to in the literature that indicate specific impacts of time spent in natural outdoor spaces to children. Boys spend more time outdoors on average than girls, the type of outdoor activity (structured vs. unstructured) can result in differing benefits, and higher amounts of outdoor time effect greater impacts [62,63]. It has also been suggested that there are geographic considerations, with green spaces around a child’s residence resulting in higher performance IQ and global IQ, yet this is not the case for blue spaces (beaches) [64,65]. These sub-groups require attention in this review, in order to capitalize on the full breadth of relevant literature but also to inform future research in this area. The potential consequences are far reaching. Furthermore, rise of obesity, rickets and asthma and decline in cardio-respiratory fitness are well documented. Richard Louv in his book ‘Last Child in the Woods’ [12] links the indoor life to an increase in attention deficit hyperactivity disorder and other mental ill-health. A report by Malone and Waite [66] found that children having fewer opportunities to explore their surrounding natural environment was hampering their social skills as well as risking stifling their long-term physical, emotional development, and wellbeing. Of additional concern, as mentioned above is the considerable body of research documenting the amount of screen time now accessed by young children and its links to developmental delay [67].

***Potential implications of the review:*** While these publications and those like them are crucial, there has not been the type of systematic evidence synthesis central to definitively supporting a change in behaviour. An exploration of the specific ways in which nature play influences a child’s communication development is essential. If outdoor spaces do have a positive impact on aspects of a child’s communication, then these opportunities can be built into school curriculum and thereby promote school readiness, engagement and overall attainment. They will also serve as initiative for new speech and language interventions. Additionally, this review has the potential to support the development of some of the most vulnerable children and families in our society, improving their happiness, health and well-being whilst also helping them to communicate. The systematic review has the potential to inform parents and schools of the impact of outdoor play, thereby encouraging local communities to development of forest school programmes and identify and cultivate wild spaces to facilitate outdoor play. Furthermore, evidence from a study such as this could support the government when considering legislation to ensure all children have access to nature.

## 2. Materials and Methods

**Review Question**: This systematic review protocol outlines the procedures for a literature review that is intended to answer the question:

What are the impacts on children’s language, communication and social skills from time spent in natural outdoor spaces?

The review will use the following definitions of language, communication and social skills in line with universal definitions within speech and language therapy: “Language skills” refers to a child’s receptive ability to understand words and sentences and expressively to use vocabulary and grammar; “Communication skills” refers to a child’s ability to use their language in conjunction with non-verbal communication skills such as eye contact, gesture and facial expression to express their thoughts and needs; and “Social skills” refers to a child’s ability to engage and maintain positive interactions with adults and other children.

This systematic review will include qualitative and quantitative data sources. The SPIDER tool for generating research questions offers a systematic review strategy for mixed-methods research studies [68] and as such will be applied to this review.

The components are in Table 1:

**Methods:** The Preferred Reporting Items for Systematic Reviews and Meta-Analyses Protocols [69] guidelines will be used for this systematic review protocol. Best practice guidance by Covidence [70] supports a systematic review approach for answering this research question. An overview of the methodology and methods used is provided in the following sections.

**Eligibility Criteria:** The international peer-reviewed literature in this systematic review will be limited to literature published between 2010–2022, which has been published in English. Quantitative, qualitative and mixed methods studies will be considered including randomised controlled trials (RCTs), case–control, cross-sectional, cohort and case studies. Chapters in books, Ph.D. theses, conference proceedings and presentations will not be included. Papers must present original data or review other empirical studies.

**Inclusion Criteria:** This systematic review is focused on the language, communication and social impacts for children from time spent in natural outdoor spaces. Included studies must report on:

- Population: Children 2–11 years.
- Spaces: outdoor areas with green space (natural vegetation such as plants, grass or trees—publicly available or forest school designated areas of school grounds) or blue spaces (water, sea, lake). NB studies that use indoor play as a comparator to outdoor play will be included but this is not essential to inclusion.
- Activities: Independent or group-based activities which may include physical activity, unstructured nature play, structured nature-based activities (crafting or gardening) or outdoor learning activities within educational settings (class lessons conducted in the outdoors). Outdoor adventure education programmes will also be included. Structured activities/outdoor interventions that specifically target speech, language and communication skills will also be accepted.
- Skills: studies must refer to aspects of a child’s language and communication development which includes parent–child interaction and social interaction. They may report on normally developing or delayed/disordered (language/communication) population.

**Exclusion Criteria:** Studies that do not fulfil all the inclusion criteria will be removed from the review.

Specific exclusion criteria:

- Population: Children over 11 and adults;
- Spaces: sports fields or courts;
- Activities: sports coaching in the outdoors;
- Skills: studies that reference cognitive skills and other health impacts of nature but not language, communication and social skills.

**Information Sources:** Academic searches will be conducted using the following electronic databases:

(1) Education Research Information Centre (ERIC);
(2) CINAHL;
(3) EMBASE;
(4) Medline;
(5) PsychINFO;
(6) The Cochrane Library;
(7) GreenFILE.

Grey literature will be accessed through Google and Google Scholar (first 100 hits) and OpenGrey (www.opengrey.eu, accessed on 9 January 2022). Relevant organisations, practitioners and researchers in the field will also be contacted to obtain information.

**Search Strategy:** Boolean operators will be used to focus the search and improve the quality, relevance and specificity of research identified. The systematic literature searching technique known as ‘pearl growing’ (also known as citation mining and snowballing) will be applied to retrieve further relevant articles.

An interdisciplinary team with a diverse range of expertise (including specialisation in speech, language and communication disorders, child psychology and outdoor learning) will be consulted in order to ensure an exhaustive and relevant search strategy. Systematic reviews and publications will be reviewed for key words and related terms will be considered. The strategy will be tested and refined until finalised and will be adapted as appropriate for each database.

**Search Terms:**Table 2 contains the search teams organised into three headings: Nature, Language and Population.

See Table 3 for search strategy for PsychINFO, such that it could be repeated.

## 3. Study Records

**Data Management:** Throughout the review process the literature management software Mendeley Desktop © will be used to manage records and data. Initial screening decisions will be made using Rayyan software [71].

**Selection Process:** In the first stage of the review, the lead researcher (SS) will initially screen the titles and abstracts of the articles identified for eligibility according to the criteria for the review. Non-English language papers, duplicate articles and articles identified as inappropriate according to the criteria of the review will be excluded. A second reviewer (JC) will independently screen 10% of titles and abstracts. Any discrepancies will be discussed, and a third reviewer (SM) will be brought in if necessary to resolve any disagreements.

In the second stage of the review, two reviewers will independently read the full text of all records which meet the eligibility criteria. Again, a third reviewer will be involved for discussion where there are disagreements between reviewers regarding eligibility of particular studies.

**Risk of Bias in Individual Studies:** The Critical Appraisal Skills Programme Checklists [72] will be used to assess risk of bias in the eligible studies. These cover both quantitative and qualitative studies. Two researchers will assess the articles against the assessment tools. The first reviewer will apply them to all included studies, and a second reviewer will independently assess risk of bias for 10% of studies to ensure consistency. Differing scores will be discussed and resolved with a third researcher. The score will be used to discuss the quality of the article only and not for inclusion or exclusion purposes.

**Data Collection Process:** The data captured from the eligible studies will be inputted into a bespoke Excel spreadsheet. In the event that information required is not available from the full text, the corresponding authors will be contacted. Communication with all external researchers will be consistent in terms of invitations to contribute and follow-up.

**Data Items:** Information in the Excel datasheet will be as follows:

- Study: Citation, author, date of publication, journal.
- Research objectives.
- Study design: methodology and methods.
- Population characteristics: age, sex, sample size, nationality of participants.
- Research space: school, garden, park, woodland.
- Activity: unstructured/structured nature play, craft, horticulture, walk, outdoor lesson.
- Outcomes related to (1) Language, inc. grammar and syntax; (2) Communication, inc parent child interaction; (3) Social skills.
- Main findings.
- Source of funding and potential conflicts of interest.

**Outcomes and Prioritisation:** Primary outcomes will include all impacts of language, communication and social skills from time spent in natural outdoor spaces.

Outcomes will include but are not limited to:

- Language: vocabulary; syntax; grammar; mean length of utterance.
- Communication and social skills: eye contact; facial expression; initiation of conversational turn; communicative responses; gesture.
- Communicative intent: questions; requests; comments; directions; affirmations; negations.
- Play: developmental level; use of imagination; interactive.
- Parent–child interaction: connectedness; responsiveness.

**Additional Outcomes:** As mentioned in the inclusion criteria, there may be studies that use play in indoor spaces as a comparator to outdoor spaces. These data may generate additional outcomes unrelated to those prioritised above.

**Data Synthesis:** Research linking nature and children’s health is diverse in definitions, methodology and design and for that reason it may be challenging to synthesise data from included studies.

It will be the heterogeneity of included studies that determines the approach to data synthesis in this systematic review. For homogenous studies, where populations, activities, outcomes and designs are sufficiently similar to make analyses appropriate, a meta-synthesis will be used to combine qualitative data and a meta-analysis for quantitative data. Statistical heterogeneity will be explored through use of appropriate statistics such as I^2^.

For studies of a heterogeneous nature a narrative synthesis will be used, with greater emphasis given to studies with a lower risk of bias and rigorous data collection/analysis approaches. This narrative synthesis will draw on the techniques of thematic analysis to categorise emergent and recurring themes within and between transcripts.

If the type and amount of data are available, a sub-group analysis will be conducted to investigate differential associations and/or effects of the following:

1. Differences by academic key stage: Early Years (EYFS) ages 2–5 years; and Key Stages 1 and 2 ages 5–11 years. Differences by gender.
2. Differences by SES.
3. Differences by ethnic group.
4. Differences by activity (structured vs. unstructured).
5. Differences by duration.
6. Differences by Type of natural outdoor space, e.g., green vs. blue, urban vs. rural.

A summary of studies will be combined into a single logic model developed by two researchers, to allow comparison and examination of how different data types relate to each other and to enable readers to identify gaps for future research.

**Confidence in Cumulative Evidence:** The Grading of Recommendations Assessment, Development and Evaluation [73] framework will be used to assess confidence in the evidence across studies at an outcome level.

## 4. Discussion

This systematic review protocol presents a clear and reproducible procedure for synthesising research currently available documenting language, communication and social impacts for children from time spent in nature. This proposed review will represent landmark research to accumulate and synthesise any associations between children’s communication abilities and time spent outdoors. The findings will provide a much-needed audit of research quality in this area, reducing risk of bias in individual studies. Additionally, it will bring together initiatives and findings from a wide range of contexts, cultures and educational and health intervention approaches, providing an international audit in this important field. It will also provide a much-needed evaluation of the definitions and impact of differing outdoor activities, thereby providing a foundation and framework for future research and the development of outdoor interventions.

As stated at the outset of this protocol, there are strong indications in the literature that time spent in natural outdoor spaces has positive impacts on many areas involved in the development of speech, language and communication skills. Attention and listening, play, lexical diversity and even the parent child interaction itself are reported to be enhanced in outdoor contexts, all of which contribute significantly to children’s communicative capabilities. It appears that the immersive environment offered by natural outdoor spaces invites the exploration, creativity and physical challenge that supports the development of these fundamentally important aspects of children’s health. It is evident that this is an area that requires attention, both in terms of a thorough examination of research already available but it is also essential that high quality studies are designed and undertaken to explore in detail the mechanisms involved. As such speech and language therapists will have the knowledge and tools to develop nature-based interventions and potentially the ability to make a simple prescription for increased time spent outdoors. Thus, proving beneficial to all children but particularly for those with speech, language and communication needs.

The systematic review is also vitally important when considering equality, diversity and inclusion. As mentioned beforehand there are inequalities in access to natural outdoor spaces. Children from low socio-economic families and black, Asian and minority ethnic families are markedly less likely to spend time outdoors than middle income families. Bearing in mind children from low socio-economic groups are also more likely to present with speech, language and communication difficulties [74], this affects a group of doubly disadvantaged children for whom greater access to natural outdoor spaces may impact in significant ways on their speech, language and communication development.

This intersectionality of disadvantage in terms of discrimination and privilege needs to be at the forefront of research in this field. The systematic review will need to consider the populations involved in these types of nature-based studies as it is likely children at the intersection of disadvantage are not represented in the groups involved in research. If this is a finding within the review it will support the development of programmes designed to include the children that will most benefit from research, outcomes and future interventions.

The review protocol is registered on PROSPERO (CRD42022316064)—the international prospective register of systematic reviews. In line with PROSPERO priorities which are focused on health-related outcomes, it is the intention of the proposed review to explore the evidence available to inform evidence-based practice in Speech and Language Therapy and support the development of nature-based interventions for the benefit of children’s health. While the systematic review process will enable the synthesis of high-quality evidence, it does not consider two important aspects of the Evidence Based Practice triangle (ASHA n.d): that of clinical expertise/expert opinion and client/patient/caregiver perspectives. The outcomes of the review will need to be integrated with clinical expertise and patient perspectives when used to inform practice.

Global consensus amongst educators, parents, paediatricians, and our mental health profession, is that children have been the invisible casualties of the COVID-19 pandemic [75,76,77,78,79]. This cohort have been severely impacted by lockdowns, school disruptions, teacher/student absenteeism and loss of social interaction, for instance. COVID-19 has affected children’s time spent outside and the documentation is amassing of reduced participation in community and sporting activities during the pandemic for our young [80]. Unquestionably, the future implications of the pandemic on children remains largely uncertain. This cohort may well become the collateral damage of the pandemic [80], and as Dickson and Gray [81] (p. 2) report for our young “their costs are very real, now and into the future. Not just their lost opportunities, but also as they bear the brunt of the debt from national economic support packages, and learn to adapt, mitigate, and manage climate change”. In short, the burdens confronting the younger generation as a result of COVID-19 are both tangible and real and require our urgent attention.

There are a number of additional limitations to this proposed systematic review. Valuable anecdotal perspectives may be lost due to the limitation on grey literature and case studies. The limitation on papers published in languages other than English may limit the scope of this systematic review and cause language bias. The exclusion of children over the age of 11 may limit a broader understanding of the impact of natural outdoor spaces on older children. It is also acknowledged that while the search terms indicated above cover an exhaustive range of the terms used in articles relating to children’s language skills and the outdoor context, it is possible that the selected criteria miss other significant key terms related to the topic area.

There may be also limitations encountered due to the wide range of methods and definitions associated with research in this area. It is hoped that the differing approaches to data synthesis will accommodate this heterogeneity in evidence. Outcomes will form a large part of the discussion and establish areas for future research, and the initiative of evidence based therapeutic interventions.

## 5. Conclusions

The systematic review set out in this paper will involve a thorough exploration of the research documenting language and communication outcomes for children from time spent in nature. Furthermore, it will provide definitions of nature-based activities and communication-based terminology, thereby guiding methods and easing synthesis and interpretation of nature play research going forward. Continuing gaps in our knowledge will inform future research and the design of those research activities.

The authors anticipate the systematic review will make a significant contribution to our understanding of this dimension of health and health inequality impacts of being outdoors for children, in line with NIHR research priorities. Research impact is potentially far reaching. Public health messages to parents describing the communicative benefits of nature play with their children, with specific information on type of play activity, nature space and dosage. Correspondingly, the findings of this review—particularly once the COVID-19 pandemic has abated—will assist in directing and informing future debate about the virtues of spending more time outdoors in natural environments. Against this backdrop, our review has the ability to inform policy makers, researchers, educators, clinicians and stakeholders within the fields of education, speech and language therapy. Finally, it will significantly contribute to key stakeholders’ ongoing dialogue around how to use natural environments to maximise communicative outcomes for all children.

## Figures and Tables

**Table 1:** Spider research question tool applied to prospective systematic review.

Sample	Children Aged 2–11 Years
Phenomenon of Interest	Impacts of time spent in natural outdoor spaces, structured or unstructured, on children’s language, communication and social skills
Design	Analysis of language Observations of communication and social skills, and parental questionnaires
Evaluation	Impacts on language, communication and social skills.
Research Type	Mixed Methods

**Table 2:** Search terms.

Nature	Language	Population
Natur*	Language Skill*	Child*
Natur* Play*	Language Development*	
Natur* Environment*	Language Delay*	
Natur* Setting*	Language Disorder*	
Natur* Exposure	Language Complex*	
Natur* School*	Grammar	
Environment* Learn*	Syntax	
Environment* Educat*	Communication	
Environment* School*	Interpersonal Communication	
Outdoor*	Communicat* Skill*	
Outdoor* Play*	Communicat* Develop*	
Outdoor* Educat*	Communicat* Delay*	
Outdoor* Learning*	Communicat* Disorder*	
Green Space*	Communicat* Intent*	
Forest School*	Social Interaction	
Forest*	Interpersonal Interaction	
Wilderness	Social Skill*	
Ecotherapy	Social Develop*	
	Social Delay*	
	Social Disorder*	
	Parent-Child Interac*	
	Parent-Child Communicat*	

* This abbreviation allows for variation in spelling and suffixes.

**Table 3:** Search strategy.

#	Database	Search Term	Results
1	PsycINFO	exp “NATURE (ENVIRONMENT)”/	3326
2	PsycINFO	(natur* ADJ2 (play* OR environment* OR setting* OR engagement OR contact* OR exposure OR school*)).ti,ab	10,661
3	PsycINFO	(environment* ADJ2 (learn* OR educat* OR school*)).ti,ab	31,319
4	PsycINFO	(outdoor*).ti,ab	6216
5	PsycINFO	(outdoor* ADJ2 (play* OR educat* OR learn*)).ti,ab	983
6	PsycINFO	(“green space*”).ti,ab	385
7	PsycINFO	(“forest school*”).ti,ab	45
8	PsycINFO	(forest* OR wilderness).ti,ab	7294
9	PsycINFO	(ecotherapy).ti,ab	42
10	PsycINFO	(1 OR 2 OR 3 OR 4 OR 5 OR 6 OR 7 OR 8 OR 9)	56,395
11	PsycINFO	exp “LANGUAGE DEVELOPMENT”/OR exp “LANGUAGE DELAY”/	31,656
12	PsycINFO	exp “LANGUAGE DISORDERS”/	40,457
13	PsycINFO	(language ADJ2 (skill* OR develop* OR complex* OR disorder* OR delay*)).ti,ab	27,823
14	PsycINFO	exp GRAMMAR/OR exp SYNTAX/	90,857
15	PsycINFO	exp COMMUNICATION/ OR exp “INTERPERSONAL COMMUNICATION”/	345,971
16	PsycINFO	(communicat* ADJ2 (skill* OR develop* OR delay* OR disorder* OR intent*)).ti,ab	21,168
17	PsycINFO	exp “SOCIAL INTERACTION”/OR exp “INTERPERSONAL INTERACTION”/	748,146
18	PsycINFO	(social ADJ2 (skill* OR develop* OR delay* OR disorder*)).ti,ab	49,225
19	PsycINFO	(“parent child” ADJ2 (interac* OR communicat*)).ti,ab	5451
20	PsycINFO	(11 OR 12 OR 13 OR 14 OR 15 OR 16 OR 17 OR 18 OR 19)	1,129,544
21	PsycINFO	(child*).ti,ab	690,338
22	PsycINFO	(10 AND 20 AND 21)	3292
23	PsycINFO	22 [DT 2010–2022]	1807

Key: # = search term number, * = truncation, searches the root of the word and any alternative endings.

## Data Availability

Not applicable.
